# Supplementary material for: Serial choriocapillaris flow changes in eyes with branched retinal vascular obstruction (BRVO)
Source: PLoS One. 2022 Nov 18;17(11):e0277988. doi: 10.1371/journal.pone.0277988 (PMC9674129; doi:10.1371/journal.pone.0277988)
Supplement: Attachment — Submitted filename: Response to Reviewers.docx [file pone.0277988.s002.docx]

**Serial choriocapillaris flow changes in eyes with branched retinal vascular obstruction (BRVO)**

**Author’s response**

November 5, 2022

Dear Editors,

Thank you for giving us the opportunity to submit a revised draft of our manuscript titled “Serial choriocapillaris flow changes in eyes with branched retinal vascular obstruction (BRVO)” to PLOS ONE. We appreciate the time and effort that you and the reviewers have dedicated to providing your valuable feedback on the manuscript. We are grateful to the reviewers for their insightful comments on the paper. We have been able to incorporate changes to reflect most of the suggestions provided by the reviewers. We have highlighted those changes within the manuscript with red color.

Here below are point-by-point responses to the reviewers’ comments and concerns.

Comments from Reviewer #1

The authors would like to thank the reviewer for taking the time to review our paper. We have made corrections and clarifications in the manuscript after going over the comments about limitations.

- Comment 1: In Methods about the statistical analysis, I think there should be the assessment of inter-oberserver reliability.

**Response**: Thank you for your thoughtful comment. The intraclass correlation coefficient (ICC) analysis was performed to calculate intergrader reliability. ICC was 0.972 for single measures and 0.986 for average measurements (p < 0.001). We clarified these contents in the revised manuscript.

- Comment 2: The structure of Table 1 and Table 2 should be modified according to the standards.

**Response**: Thank you for your thoughtful comments. We respected your opinion, and we made a modification of the table contents to better fit the standards.

- Comment 3: In Discussion, the structure needs some adjustments, for the logic is confusing to some extent.

**Response**: We acknowledge that the explanation and logics we made seemed to cause confusion. We corrected the discussion contents to be more understandable. Thank you.

We look forward to hearing from you in due time regarding our submission and to responding to any further questions and comments you may have.

Sincerely,

Young-Hoon Park, MD, PhD

Department of Ophthalmology and Visual Science, Seoul St. Mary's Hospital, College of Medicine, The Catholic University of Korea, 222 Banpo-daero, Seocho-gu, Seoul 06591, Republic of Korea

E-mail: parkyh@catholic.ac.kr

Tel : 82-2-2258-6201

Fax : 82-2-599-7405
